# Randomized Clinical Trial of the Innovative Bilayered Wound Dressing Made of Silk and Gelatin: Safety and Efficacy Tests Using a Split-Thickness Skin Graft Model

**DOI:** 10.1155/2015/206871

**Published:** 2015-07-05

**Authors:** Sukhontha Hasatsri, Apichai Angspatt, Pornanong Aramwit

**Affiliations:** ^1^Bioactive Resources for Innovative Clinical Applications Research Unit and Department of Pharmacy Practice, Faculty of Pharmaceutical Sciences, Chulalongkorn University, Phayathai Road, Pathumwan, Bangkok 10330, Thailand; ^2^Division of Plastic & Reconstructive Surgery, Department of Surgery, Faculty of Medicine, Chulalongkorn University, Phayathai Road, Pathumwan, Bangkok 10330, Thailand

## Abstract

We developed the novel silk fibroin-based bilayered wound dressing for the treatment of partial thickness wounds. And it showed relevant characteristics and accelerated the healing of full-thickness wounds in a rat model. This study is the clinical evaluation of the bilayered wound dressing to confirm its safety and efficacy for the treatment of split-thickness skin donor sites. The safety test was performed using a patch model and no evidence of marked and severe cutaneous reactions was found. The efficacy test of the bilayered wound dressing was conducted on 23 patients with 30 split-thickness skin graft donor sites to evaluate healing time, pain score, skin barrier function, and systemic reaction in comparison to Bactigras. We found that the healing time of donor site wounds treated with the bilayered wound dressing (11 ± 6 days) was significantly faster than those treated with Bactigras (14 ± 6 days) (*p* = 10^−6^). The wound sites treated with the bilayered wound dressing showed significantly less pain and more rapid skin functional barrier recovery than those treated with Bactigras (*p* = 10^−5^). Therefore, these results confirmed the clinical safety and efficacy of the bilayered wound dressing for the treatment of split-thickness skin graft donor sites.

## 1. Introduction

Split-thickness skin grafting is a surgical procedure that harvests the healthy skin and uses it to cover the wound to activate wound healing. The site where the healthy skin is removed is called the split-thickness skin graft donor site, which is a type of partial thickness wound. The appropriate management of split-thickness graft donor sites is important to promote healing and to control the pain and comfort of the patients. Paraffin gauze dressings (e.g., Bactigras and Sofra-tulle) are used for the treatment of this donor site wound. However, they are sometimes very adhesive to the wound surface and do not absorb much wound exudate [1–3].

Previously, we developed the novel silk fibroin-based bilayered wound dressing [4]. The bilayered wound dressing is composed of a wax-coated silk fibroin woven fabric as a nonadhesive layer and a sponge made of sericin and glutaraldehyde-cross-linked silk fibroin/gelatin as a bioactive layer. The* in vitro* analysis showed that the wax-coated silk fibroin fabric had improved mechanical properties and adhered less to the wound, while the spongy bioactive layers had a homogeneous porous structure and controllable biodegradation and supported the attachment and proliferation of L929 mouse fibroblasts. We also tested this bilayered wound dressing* in vivo* and proved that it could promote healing of the full-thickness wounds in a rat model by the induction of epithelialization and collagen formation [4].

Following this study, the physical and biological assessments of this bilayered wound dressing were systematically performed to evaluate its efficacy for clinical applications [5]. We have recently reported that the bilayered wound dressing showed a continuous absorption rate of wound exudate and good conformability and allowed for dehydration to control moisture levels. Furthermore, in terms of biological activities, the bilayered dressing was not toxic to skin cells but promoted cell migration and collagen production. Based on these data, a bilayered wound dressing would be a promising choice for wound therapy. To prove this, a clinical trial of a bilayered wound dressing is necessary.

This study therefore investigates the close-to-market stage of the innovative bilayered wound dressing that was previously developed for the treatment of partial thickness wounds using split-thickness skin graft donor sites as a model. The safety test of the bilayered wound dressing was performed on 110 healthy volunteers using a patch model to evaluate cutaneous reactions and skin irritation. The efficacy test of the bilayered wound dressing was conducted on 23 patients with 30 split-thickness skin graft donor sites to evaluate healing time, pain score, skin barrier function (transepidermal water loss), wound infection, systemic adverse reactions, morphology of epithelial cells, and blood biochemistry. The safety and efficacy of our bilayered wound dressing were compared to Bactigras (a standard wound dressing for donor site wound treatment) in a prospective, randomized, and controlled match pair trial. The results from this study could support the use of bilayered wound dressings for split-thickness skin donor site or any partial thickness wound treatments in the future.

## 2. Materials and Methods

### 2.1. Materials

The bilayered wound dressing was prepared according to the previously developed technique [4]. In this study, the combination of silk fibroin, silk sericin, and gelatin was used to prepare the wound dressing material. Silk fibroin and gelatin solutions at the mixing ratio of 20 : 80 were mixed with 1% w/w sericin solution to obtain a final solution concentration at 4% w/w and then cross-linked with glutaraldehyde (0.02% v/v). The mixture was cast onto the silk fabric and incubated at 4°C for 24 h to allow the cross-linking reaction. The residual aldehyde groups of glutaraldehyde in the cross-linked gels were removed by glycine solution, followed by washing the gels repeatedly with deionized water. The gels were freeze-dried to obtain the bilayered wound dressing (thickness = 0.4 cm). To increase flexibility, the bilayered wound dressing was immersed in glycerin (20% v/v) at room temperature for 4 h, followed by air-drying for 10 h. The bilayered wound dressing was sterilized by gamma irradiation before use. Bactigras, which is a medicated chlorhexidine paraffin gauze dressing, was purchased from Smith & Nephew (London, United Kingdom).

### 2.2. Peel Test with Porcine Skin

A full-thickness wound (1-cm depth) was created on porcine skin that was obtained within 2 h after sacrifice. The bilayered wound dressing and Bactigras were randomly attached to the wounds. After 12 h, the dressings were removed from wounds and the number of cells attached to the dressings was analysed by fluorometric quantification of cellular DNA according to the method reported by Takahashi et al. [6]. The number of cells indicated the adhesiveness of the wound dressing.

### 2.3. Clinical Safety Test of the Bilayered Wound Dressing in Healthy Volunteers

The test was conducted from November 2012 to February 2013 at the Department of Pharmacy Practice, Pharmaceutical Sciences, Chulalongkorn University, Bangkok, Thailand. The study was approved by the ethics committee of the Faculty of Pharmaceutical Sciences (Protocol review number 12-33-013).

#### 2.3.1. Participants

A total of 110 healthy volunteers were recruited in this study. Inclusion criteria included the following: (1) signed informed consent, (2) age between 18 and 65 years, and (3) normal physical and neurological examinations. Exclusion criteria included the following: (1) use of immunosuppressive drugs and antihistamines within the last 2 weeks, (2) skin diseases such as psoriasis and infectious dermatological conditions, and (3) immunodeficiency diseases. The demographic data of healthy volunteers are shown in Table 1.

#### 2.3.2. Study Protocol

The study was a prospective, randomized, controlled, and matched pair trial. Eligible patients were given verbal and written information about the study and were provided written consent. Then, the baseline characteristics were recorded. The patch test consisted of four phases: (1) beginning phase, (2) induction phase I, (3) induction phase II, and (4) challenge phase [7].

First, the back of the healthy volunteers was divided into two sites. Skin irritation assessments including skin redness (erythema level) and skin darkness (melanin level) were performed and reported in phase 1. Then, each site was randomized to receive the bilayered wound dressing or Bactigras (2 × 2 cm^2^) covered with a self-adhesive nonwoven fabric. Both dressings were left on the skin for 3 days. On the second visit (3 days after phase 1), both dressings were changed and left for additional 3 days. Skin assessments were performed prior to replacing a new wound dressing to obtain data from induction phase I. On the third visit (3 days after the induction phase I), both dressings were removed and skin assessments were performed to obtain data from induction phase II. On the fourth visit (7–10 days after the induction phase II), both dressings were applied on identical areas and left for an additional 3 days. On the fifth visit, both dressings were removed and skin assessments were performed to obtain data from the challenge phase.

The skin assessment was performed using a Mexameter MX 18 (Courage + Khazaka Electronic GmbH, Germany) and photographs of the skin were taken within 30 min after the dressings were removed. All skin photographs were evaluated for any visual skin irritation using the repeated insult patch test (RIPT) scale by 2 clinical dermatologists without identifying each dressing (a double-blinded study) [8].

### 2.4. Clinical Efficacy Test of the Bilayered Wound Dressing in the Treatment of Split-Thickness Skin Graft Donor Sites

The study was conducted from October 2013 to December 2014 at the Division of Plastic and Reconstructive Surgery, Department of Surgery, King Chulalongkorn Memorial Hospital, Bangkok, Thailand. The study was in compliance with the latest revision of the Declaration of Helsinki and was approved by the institutional review board of the Faculty of Medicine, Chulalongkorn University, Bangkok, Thailand (IRB number 184/56; Clinical Trial Registration number NCT02091076).

#### 2.4.1. Participants

A total of 30 donor sites from 23 patients who underwent a split-thickness skin graft procedure were recruited for this study. There were 6 patients with 2 donor site wounds and each patient received 2 dressings of each kind. Inclusion criteria included (1) signed informed consent, (2) age between 18 and 65 years, and (3) donor sites located on the thigh. Exclusion criteria included (1) immunocompromised patients, (2) diabetes mellitus, (3) psychiatric disorders or physical disabilities, and (4) low serum albumin level (less than 3.0 g/dL) [9]. The demographic data of patients who underwent a split-thickness skin graft are shown in Table 1.

#### 2.4.2. Study Protocol

The study was a prospective, randomized, controlled, and match pair trial. The eligible patients were given verbal and written information about the study and were provided written consent. Then, the baseline characteristics were recorded. A split-thickness skin graft donor site was taken from the thigh with a Zimmer dermatome, which can be adjusted to the setting of the electrodermatome by rotating the level indicated on the instrument, and applied with epinephrine soaked gauze until surgery was completed. Next, the bilayered wound dressing and Bactigras were randomly applied to the split-thickness skin graft donor site. The patients did not know which dressing was bilayered wound dressing or Bactigras but they could be easily identified by the surgeon when the dressing was changed (i.e., a single-blinded study). Photographs of the split-thickness skin graft donor site were taken to measure the donor site area using ImageJ software.

**Figure 1:**
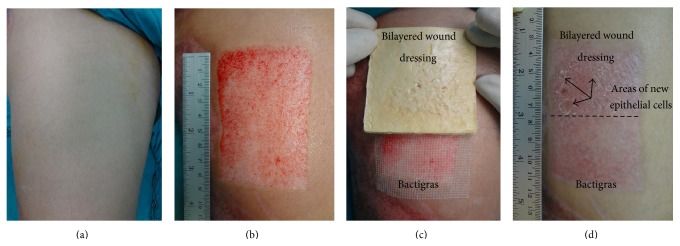
(a) Thigh area before surgery, (b) split-thickness skin graft donor site after the skin was taken, (c) split-thickness skin graft donor site treated with Bactigras and bilayered wound dressing, and (d) healed split-thickness skin graft donor site (postdonor site healing day 1 for the site treated with bilayered wound dressing and postdonor site healing day 0 for the site treated with Bactigras).

The split-thickness skin graft donor site was divided into a cephalad half and a caudal half of equal size, and each site was randomized to receive the bilayered wound dressing or Bactigras (Figure 1). Then, both dressings were covered with gauze pads and elastic bandages. The dressings were not changed, except when they were fully soaked with exudate, when they had peeled off, or when there was any sign of infection.

The healing time was recorded from the time of no exudates or pain to when the dressing spontaneously peeled off from the donor site [3]. Local pain was evaluated according to a visual analogue scale from 0 (no pain) to 10 (unbearable pain) points and was recorded on days 1 to 5 postoperatively. During pain assessment, the donor site wounds were covered with gauze pads and rolls which could not be identified by either surgeon or patient (i.e., double-blinded study). Skin barrier function recovery of the donor site was evaluated by measuring transepidermal water loss (TEWL: water vapour flux density diffusing from the skin to the external environment) [10] using a Tewameter TM 300 (Courage + Khazaka Electronic GmbH, Germany). TEWL was measured on the day that the wound was found to be completely healed, after 1 week, and from 1 to 5 months later. Wound infection was based on signs of infection (i.e., redness, swelling, inflammation, purulent exudate, or malodour) and body temperature. Systemic adverse reactions were observed from liver and renal functions between pre- and postoperation (1–3 days).

### 2.5. Statistical Analysis

All qualitative data are represented as frequencies and percentages. All quantitative data are shown as the mean ± standard deviation and median ± interquartile range. Repeated measures ANOVA was used to compare the erythema and melanin levels during each phase. The Wilcoxon signed rank test was used to compare the healing time, pain level, and TEWL between the dressing groups. The McNemar test was used to compare laboratory data between pre- and postoperative days. The data were analysed using SPSS version 22.0 software, and a value of *p* < 0.05 was considered to be statistically significant.

## 3. Results

### 3.1. Adhesiveness of the Bilayered Wound Dressing

From the peel test on the full-thickness wound of porcine skin, we found that the bilayered wound dressing could be removed from the wound much more easily than the Bactigras. Some cells were found on Bactigras (~21 × 10^4^ cells/dressing) while no cells were observed on the bilayered wound dressing. The large number of cells left on the dressing indicated the damage of new epithelial cells upon removal.

### 3.2. Clinical Safety of the Bilayered Wound Dressing

Baseline characteristics of healthy volunteers for the patch test are summarized in Table 1. The healthy volunteers consisted of 28.2% males and 71.8% females with an average age of 39.9 years. The average weight, height, and BMI of all volunteers were 60.1 kg, 160.5 cm, and 23.3 kg/m^2^, respectively.  Table 2 shows the erythema and melanin levels of each phase after application of the Bactigras or bilayered wound dressing. The results indicate that the erythema and melanin levels at the beginning phase (238 ± 83 units for erythema and 230 ± 99 units for melanin) were significantly higher than those at the induction phase I, induction phase II, and challenge phase (~225 ± 79 units for erythema and 220 ± 97 units for melanin) for both dressings. There was no significant difference in erythema and melanin levels between induction phases I and II and the challenge phase. Most volunteers (98.6% for Bactigras and 95.9% for the bilayered wound dressing) showed no evidence of any effect on the skin after the patch test. However, there was evidence of mild and moderate erythema (1.8%) on the skin patched with the bilayered wound dressing. Minimal faint (light pink) uniform or spotty erythema was observed on 1.4% of the skin patched with Bactigras and 0.5% of the skin patched with the bilayered wound dressing. There was no evidence of marked and severe responses on the skin of any volunteer.

### 3.3. Clinical Efficacy of the Bilayered Wound Dressing

The baseline characteristics of patients who underwent a split-thickness skin graft procedure are summarized in Table 1. The number of male and female patients was equal. The average age, weight, height, and BMI of all patients were 37.3 years old, 58.5 kg, 162.2 cm, and 22.2 kg/m^2^, respectively. The size and thickness of the split-thickness skin graft donor sites of all patients were approximately 107 ± 43 cm^2^ and 242 ± 34 *μ*m, respectively.

**Figure 2:**
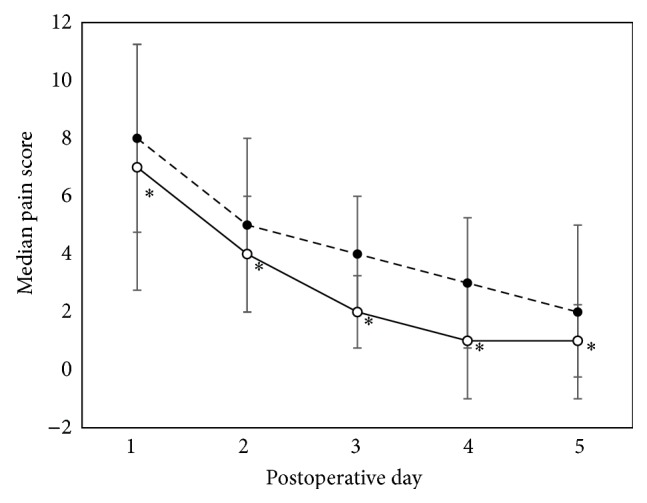
Median pain score of donor site wound treated with Bactigras and bilayered wound dressing. (- -●- -) Bactigras, (- -○- -) bilayered wound dressing.  ^*∗*^Significant difference (*p* < 0.001 versus Bactigras), calculated by a Wilcoxon signed rank test.

Table 3 presents the time that the split-thickness skin graft donor sites treated with Bactigras or the bilayered wound dressing had completely healed. The healing time of donor site wounds treated with the bilayered wound dressing (11 ± 6 days) was significantly faster than those treated with Bactigras (14 ± 6 days).  Figure 1(d) shows the appearance of the healed donor site. Epithelium regeneration was observed on the site treated with the bilayered wound dressing, as compared to the site treated with Bactigras.  Figure 2 shows pain scores of donor site wounds treated with Bactigras or the bilayered wound dressing for 1–5 days postoperatively. Pain scores of the wounds treated with both dressings were gradually reduced from day 1 to day 5. On each day, pain scores of wounds treated with the bilayered wound dressing were significantly lower than those of wounds treated with Bactigras.

**Figure 3:**
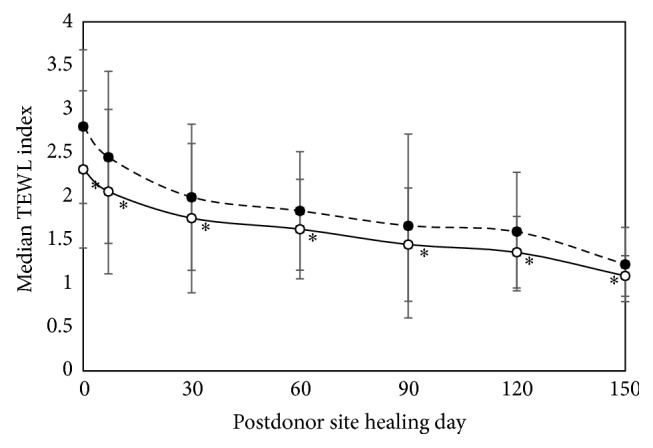
Median transepidermal water loss (TEWL) of healed donor site treated with Bactigras and bilayered wound dressing. (- -●- -) Bactigras, (- -○- -) bilayered wound dressing. TEWL index = TEWL of postdonor site healing day A/TEWL of adjacent normal skin day A.  ^*∗*^Significant difference (*p* < 0.001 versus Bactigras), calculated by a Wilcoxon signed rank test.

Figure 3 shows the median TEWL of the healed donor site treated with Bactigras or the bilayered wound dressing. Median TEWL of the wounds treated with Bactigras and the bilayered wound dressing on the day of donor site healing (day 0) were 2.8 ± 0.8 and 2.3 ± 0.9 times, respectively, higher than that of adjacent normal skin. Additionally, the median TEWL on donor site after 150 days was 1.22 ± 0.43 and 1.09 ± 0.23 times significantly higher than adjacent normal skin for Bactigras (*p* = 10^−6^) and the bilayered wound dressing (*p* = 10^−6^), respectively. As with the postdonor site healing days, the donor site wounds treated with the bilayered wound dressing showed a significantly lower TEWL index than those treated with Bactigras (*p* = 10^−5^), implying a faster TEWL recovery of the wounds treated with the bilayered wound dressing.

There were no signs of donor site infection in the wounds treated with either wound dressing. The median body temperatures at 1–5 days postoperatively were lower than 37°C, indicating no fever in any patients.  Table 4 presents the blood biochemistry, which indicated systemic adverse reactions in patients 1–3 days after operation. At 1–3 days postoperatively, the values of blood urea nitrogen (BUN), serum creatinine (SCr), aspartate aminotransferase (AST), alanine aminotransferase (ALT), and alkaline phosphatase (ALP) were not much different (or even lower), compared to the preoperative values. Most values were found to be in a normal range. There was no sign of systemic reaction on any patients.

## 4. Discussion

The split-thickness skin graft donor site is a type of sterile surgical wound that usually has a high amount of wound exudate. In Thailand, paraffin gauze dressing with antibacterial agent (e.g., Bactigras) is commonly used for the treatment of donor site wounds, due to the climate in Thailand. However, this type of dressing does not absorb much exudate and it is sometimes highly adhesive to the wound surface, resulting in pain during application and removal [1–3]. In our previous work, we developed the innovative silk fibroin-based bilayered wound dressing that was designed with a spongy structure to increase the absorption of wound exudate and a nonadhesive wound contact layer to reduce adherence at the wound surface [4].

In this study, a clinical trial of the bilayered wound dressing was conducted with healthy volunteers and patients who underwent a split-thickness skin graft procedure to evaluate the safety and efficacy of the novel bilayered wound dressing, as compared to the clinically used wound dressing, Bactigras. A patch test was used to measure skin irritation in healthy volunteers. The results indicated that the redness and darkness of skin patched with either wound dressing did not increase from baseline and no marked and severe cutaneous reactions appeared in any volunteer (Table 2). This may be because all components of the bilayered dressing are naturally derived and biocompatible. The skin redness and darkness before and after application were found to be comparable to those reported by Maenthaisong et al. [11]. These results confirm that the bilayered wound dressing is safe for clinical use.

The bilayered wound dressing and Bactigras were used as dressings for the treatment of split-thickness skin graft donor sites. The healing of split-thickness skin graft donor site wounds indicated the efficacy of the wound dressing. The split-thickness skin graft donor site wounds treated with the bilayered wound dressing healed faster than the wounds treated with Bactigras (Table 3), possibly because proteins such as silk fibroin, gelatin, and sericin in the bilayered wound dressing are bioactive and could induce migration, adhesion, proliferation, and tissue regeneration [12–18]. The ability of silk fibroin to promote adhesion and proliferation of epidermal cells and promote wound healing has been widely reported [19–21]. Sugihara et al. showed that silk fibroin films could heal the full-thickness skin wounds in rats at a faster rate than the traditional porcine-based wound dressings because the silk fibroin film had more potential to promote epithelialization and showed low inflammatory response [22, 23]. Baoyong et al. reported that the membrane made of recombinant spider silk protein promoted the recovery of wound skin by increasing the expression and secretion of basic fibroblast growth factor and hydroxyproline [24].

Gelatin is a denatured collagen, which is the main component of skin and other connective tissue. It is known that gelatin molecules contain a number of functional groups that promote cellular activities. Some gelatin-based materials have been successfully used as wound dressings to promote wound healing [25, 26]. In addition, sericin added to the silk fibroin/gelatin sponge has been shown to promote skin cell proliferation, collagen production, and wound healing [12, 13]. All of these bioactive properties of the material would explain why the bilayered wound dressing accelerated the healing of split-thickness skin graft donor site wounds. In addition to the accelerated healing time, the split-thickness skin graft donor site wounds treated with the bilayered wound dressing showed less pain than those treated with Bactigras (Figure 2), possibly due to the reduced adhesion of the wax-coated silk fibroin woven fabric layer of the bilayered wound dressing, which might minimize the disruption of the reepithelialized surface.

Furthermore, we demonstrated that the donor sites treated with the bilayered wound dressing had more rapid skin functional barrier recovery (which is considered to be the endpoint of wound healing) than those treated with Bactigras (Figure 3). This observation also indicates the efficacy of the bilayered wound dressing at wound healing. Finally, the safety of the wound dressings was verified in terms of systemic effects by confirming that indicators of toxicity such as BUN, SCr, AST, ALT, and ALP and body temperature seemed to be in their normal ranges (Table 4). However, there was a slight decrease in serum albumin levels, possibly due to the loss of albumin through the donor site wound created after harvesting the skin grafts [18]. This result confirmed that there was no evidence of abnormal renal or hepatic functions or donor site infections. Although these data provide good clinical support for the use of the bilayered wound dressing, some weaknesses of this study include the small number of wounds investigated as well as the evaluation of pain levels. Some patients did not experience pain on the first postoperative day. As a result, the pain scores of wounds treated with the bilayered wound dressing were estimated to be equal to those treated with Bactigras. Moreover, some parts of the clinical experiment could not avoid being single-blinded due to the characteristics of each wound dressing.

## 5. Conclusions

This clinical investigation confirmed the safety and efficacy of the bilayered wound dressing for the treatment of split-thickness skin graft donor sites. The bilayered wound dressing is thus recommended as an option for split-thickness skin donor sites or other partial thickness wounds treated in the clinic.

## Figures and Tables

**Table 1:** Demographic data of healthy volunteers and patients who underwent a split-thickness skin graft procedure for patch test and donor site treatment, respectively.

	Healthy volunteers	Patients who underwent a split-thickness skin graft procedure
	Number or mean ± SD (range)	Number (%) or mean ± SD (range)
Sex		
Male	31	15
Female	79	15
Age (years)	39.9 ± 12.8 (20–61)	37.3 ± 15.0 (18–64)
Weight (kg)	60.1 ± 12.4 (41–96)	58.5 ± 12.3 (42–91)
Height (cm)	160.5 ± 8.1 (140–182)	162.2 ± 7.3 (149–179)
BMI (kg/m^2^)	23.3 ± 4.2 (16.5–37.1)	22.2 ± 4.4 (15.6–32.8)

**Table 2:** Erythema and melanin levels of healthy volunteers' skin at the beginning phase, induction phase I, induction phase II, and challenge phase.

	Beginning phase	Induction phase I	Induction phase II	Challenge phase
Erythema level				
Bactigras	233.57 ± 81.96	218.93 ± 78.44^*∗*^	217.31 ± 78.49^*∗*^	219.45 ± 75.60^*∗*^
Bilayered wound dressing	243.70 ± 85.09	233.17 ± 83.47^*∗*^	230.86 ± 82.75^*∗*^	231.82 ± 79.91^*∗*^

Melanin level				
Bactigras	226.47 ± 97.65	216.22 ± 95.03^*∗*^	216.30 ± 95.13^*∗*^	216.28 ± 95.09^*∗*^
Bilayered wound dressing	234.89 ± 101.84	224.64 ± 100.17^*∗*^	224.69 ± 99.93^*∗*^	224.64 ± 99.97^*∗*^

^*∗*^Significant differences (*p* < 0.001 versus beginning phase), calculated by repeated measures ANOVA.

**Table 3:** The median healing time of split-thickness skin graft donor sites in each dressing.

The healing time of split-thickness skin graft donor sites (days)	
	Median ± IQR (range)
Bactigras	14.0 ± 6.0 (9–19)
Bilayered wound dressing	11.0 ± 6.0 (7–18)^*∗*^

^*∗*^Significant difference (*p* < 0.001 versus Bactigras), calculated by a Wilcoxon signed rank test.

**Table 4:** Blood biochemistry of patients at preoperative and postoperative days of split-thickness skin grafts.

Parameter^a^	Preoperative day	Postoperative day (1–3 days)	*p* value^b^
	Median ± IQR (range)	Median ± IQR (range)	
Renal functions			
BUN (mg/dL) (normal value: 7–20)	13.00 ± 4.25 (4–26)	10.00 ± 2.50 (4–24)	<0.001
SCr (mg/dL) (normal value: 0.50–1.00)	0.80 ± 0.17 (0.50–1.00)	0.70 ± 0.20 (0.50–1.00)	0.388
Hepatic functions			
AST (U/L) (normal value: 5–35)	19.00 ± 10.25 (12–87)	16.00 ± 9.25 (11–60)	0.022
ALT (U/L) (normal value: 0–40)	24.00 ± 10.25 (4–66)	10.50 ± 9.25 (5–46)	<0.001
ALP (U/L) (normal value: 40–120)	80.00 ± 51.00 (46–163)	71.00 ± 53.25 (35–161)	<0.001
Albumin (g/dL) (normal value: 3.0–5.0)	3.60 ± 1.30 (3.0–4.8)	3.20 ± 0.93 (2.2–4.2)	<0.001

^a^BUN: blood urea nitrogen; SCr: serum creatinine; AST: aspartate aminotransferase; ALT: alanine aminotransferase; ALP: alkaline phosphatase.

^b^Calculated by a Wilcoxon signed rank test to compare the difference of each laboratory pre- and postoperative day.
